# The whole-body motor skills of children with autism spectrum disorder taking goal-directed actions in virtual reality

**DOI:** 10.3389/fpsyg.2023.1140731

**Published:** 2023-04-06

**Authors:** Maria Eleonora Minissi, Lucía Gómez-Zaragozá, Javier Marín-Morales, Fabrizia Mantovani, Marian Sirera, Luis Abad, Sergio Cervera-Torres, Soledad Gómez-García, Irene Alice Chicchi Giglioli, Mariano Alcañiz

**Affiliations:** ^1^Instituto Universitario de Investigación en Tecnología Centrada en el Ser Humano (HUMAN-tech), Universitat Politécnica de Valencia, Valencia, Spain; ^2^Centre for Studies in Communication Sciences “Luigi Anolli” (CESCOM), Department of Human Sciences for Education “Riccardo Massa”, University of Milano - Bicocca, Milan, Italy; ^3^Red Cenit, Centros de Desarrollo Cognitivo, Valencia, Spain; ^4^Facultad de Magisterio y Ciencias de la Educación, Universidad Católica de Valencia, Valencia, Spain

**Keywords:** autism spectrum disorder, children, motor skills, body tracking, virtual reality, virtual interaction

## Abstract

Many symptoms of the autism spectrum disorder (ASD) are evident in early infancy, but ASD is usually diagnosed much later by procedures lacking objective measurements. It is necessary to anticipate the identification of ASD by improving the objectivity of the procedure and the use of ecological settings. In this context, atypical motor skills are reaching consensus as a promising ASD biomarker, regardless of the level of symptom severity. This study aimed to assess differences in the whole-body motor skills between 20 children with ASD and 20 children with typical development during the execution of three tasks resembling regular activities presented in virtual reality. The virtual tasks asked to perform precise and goal-directed actions with different limbs vary in their degree of freedom of movement. Parametric and non-parametric statistical methods were applied to analyze differences in children’s motor skills. The findings endorsed the hypothesis that when particular goal-directed movements are required, the type of action could modulate the presence of motor abnormalities in ASD. In particular, the ASD motor abnormalities emerged in the task requiring to take with the upper limbs goal-directed actions with low degree of freedom. The motor abnormalities covered (1) the body part mainly involved in the action, and (2) further body parts not directly involved in the movement. Findings were discussed against the background of atypical prospective control of movements and visuomotor discoordination in ASD. These findings contribute to advance the understanding of motor skills in ASD while deepening ecological and objective assessment procedures based on VR.

## 1. Introduction

Autism spectrum disorder (ASD) is a neurodevelopmental disorder characterized by impairments in social interaction and communication, and restrictive and repetitive behaviors and interests (American Psychiatric Association, 2013). ASD is characterized by a high prevalence rate (1 out 100 children worldwide; World Health Organization, 2022) and shows significant variability in the clinical phenotype, which has led clinicians to differentiate three ranges of severity within the spectrum (American Psychiatric Association, 2013).

Since early childhood, the variety of the disorder etiopathogenesis is evident, and some symptoms appear even at 6 months of age (Jones and Klin, 2013). However, ASD is usually diagnosed much later leading, in turn, to delayed access to the intervention system, which can hamper potential treatment outcomes (Clark et al., 2018; Vant Hof et al., 2021). In addition, the ASD diagnosis is typically based on semi-structured procedures with limited objectivity and ecological validity, which may affect the reliability of the assessments (Goldstein and Ozonoff, 2018; Alcañiz Raya et al., 2020a,b; Alcañiz et al., 2022). Therefore, there is a need to anticipate the ASD diagnosis as much as possible, employing standardized and objective measurements in ecological settings (Zwaigenbaum et al., 2013; Bölte et al., 2016; Minissi et al., 2021, 2022).

Over the last decade, the scientific community has focused on investigating potential ASD biomarkers detectable in early childhood, eventually boosting the publication of studies on the topic (Bölte et al., 2016; Bridgemohan et al., 2019). There are many instances of ASD biomarkers appearing in early childhood that are related to the nuclear symptoms of the disorder (Wetherby et al., 2004; Zwaigenbaum et al., 2005; Watt et al., 2008; Ozonoff et al., 2010). Furthermore, motor abnormalities are reaching consensus as a promising ASD biomarker, regardless of the level of symptom severity and the children’s age (Fournier et al., 2010; Zwaigenbaum et al., 2013; Bhat, 2020, 2021; Zampella et al., 2021). Indeed, in some cases, the motor abnormalities related to ASD are evident even in early childhood when the disorder has not been detected yet (i.e., 4 to 6 months of age; Teitelbaum et al., 1998). Kanner (1943) was the first one to theorize that motor abnormalities are an essential feature of ASD, describing the existence of gross motor incoordination in this specific population. Subsequently, a link between motor abnormalities and the main symptoms of ASD was found (Leary and Hill, 1996), suggesting researchers deepen their knowledge of this potential symptom domain. The motor abnormalities represent deficits concerning gross movements, such as whole-body coordination (McPhillips et al., 2014; Kaur et al., 2018), postural control (Lim et al., 2017; Stins and Emck, 2018), and visuomotor coordination (Dewey et al., 2007; Fleury et al., 2013). Similarly, fine movements are impaired in individuals with ASD, such as dexterity (Kaur et al., 2018; Khoury et al., 2020), handwriting/drawing (Kushki et al., 2011; Fleury et al., 2013), and object manipulation (Crippa et al., 2015; Vabalas et al., 2020). In particular, previous studies on individuals with ASD reported, among others, postural instability, unnecessary movements in the whole body, and atypical movements in the upper limbs while performing certain actions (e.g., Forti et al., 2011; Lim et al., 2017; Alcañiz Raya et al., 2020b; Simeoli et al., 2021; Zhao et al., 2022). A study by Staples and Reid (2010) showed that the motor skills of children with ASD are similar to those of children with typical development (TD) approximately half their age.

To date, the risk for motor abnormalities in individuals with ASD is 22 times greater than in the population with TD. This risk increased 5 to 5,7 times more as symptom severity increased in repetitive behaviors and social behavior domains, respectively (Bhat, 2021). Such prevalence may suggest a specific nature of motor abnormalities in ASD and the need to recognize the motor abnormalities as diagnostic criteria or at least as specifier of ASD (Miyahara, 2013; Bhat, 2020; Licari et al., 2020).

In addition to symptom-based classifications as the Diagnostic and Statistical Manual of Mental Disorders (DSM; American Psychiatric Association, 2013), the Research Domain Criteria (RDoC) project proposed by the National Institute of Mental Health (NIMH) is reaching consensus in providing a biological-based framework of the mental disorders. The purpose of RDoC is describing the functioning in mental health disorders (like ASD) at neurobiological level across different domains. Recently, the sensorimotor domain has been inserted as new cluster to be studied in psychopathology, due to the consistent evidence of motor deficits in mental health disorders (National Institute of Mental Health, 2019). In particular, recent evidence in ASD research suggested that motor features are as sensitive as further RDoC features in objectively classifying the disorder (Harrison et al., 2021). Interestingly, the motor features improved the accuracy of ASD classification based on the further RDoC domains.

The motor abnormalities in ASD may be linked to deficits in the prospective control of movements, which is required to transform individual intentions efficiently into motor operations (Klin et al., 2003; Cattaneo et al., 2007; Trevarthen and Delafield-Butt, 2013; Crippa et al., 2015). In this process, the mirror neuron system plays an essential role in fostering the planning of actions. There is evidence that in children with ASD, there is a dysfunction in the mirror neuron system, which provokes deficits in the mental organization of motor actions (Oberman et al., 2005; Martineau et al., 2008; Fabbri-Destro et al., 2009). In particular, individuals with ASD endeavor to make good fine motor acts because they mentally manage the action submovements as independent rather than part of a whole (Fabbri-Destro et al., 2009).

The research methods on ASD motor abnormalities initially employed the qualitative analysis of motor behaviors (e.g., Kanner, 1943; Teitelbaum et al., 1998), as well as the application of standardized behavioral coding scales (e.g., Adrien et al., 1993; Ozonoff et al., 2008). However, the qualitative observation made by an expert may not precisely quantify the complexity of motor movements (Zhao et al., 2022). Consequently, recent technological advances have moved forward the objective and quantitative monitoring of motor biomarkers in ASD, using wearable sensors, such as motion trackers and accelerometers, and kinematic analysis, such as 3D motion capture (Wilson et al., 2018). Most recent studies focused on quantitatively detecting one motor domain at a time rather than whole-body movements. Indeed, previous studies mainly described the fine motor movements of particular body parts (i.e., the lower/upper limbs and head) during the execution of specific actions (e.g., Forti et al., 2011; Crippa et al., 2015; Hasan et al., 2017; Dawson et al., 2018; Vabalas et al., 2020; Simeoli et al., 2021). However, how the whole-body movements are impaired in children with ASD during the execution of specific actions and whether body parts are driving the effect is still unclear.

To our knowledge, kinematic analyses have only been scarcely used to quantitatively assess whole-body motor abnormalities in infants diagnosed with ASD. These studies identified ASD automatically with considerable precision, even though they were characterized by small sample sizes (e.g., Ardalan et al., 2019; Alcañiz Raya et al., 2020b; Kojovic et al., 2021; Zhao et al., 2022). Findings showed that, compared to children with TD, children with ASD presented more variability and entropy in their motor actions, as well as excessive and less complex movements. These studies represent promising attempts to ASD classification based on the motor movements of the whole body and join the vast literature on the topic demonstrating the relevance of motor discoordination to make ASD diagnosis. Nevertheless, besides the need to include motor abnormalities as ASD diagnostic criteria or specifier, there is still the need to describe motor abnormalities objectively in early childhood so that clinicians may start to recognize them. Their recognition would improve the access to physical/motor therapy in autistic children, in addition to the traditional intervention system (Teitelbaum et al., 1998; Green et al., 2009; Bhat, 2021). Indeed, there is little evidence that physical/motor therapy in individuals with ASD may improve core symptoms besides motor abnormalities, such as social skills (Bremer and Lloyd, 2016; Ketcheson et al., 2017).

The present study deepens this question by exploring whole-body motor skills of children with ASD during the execution of precise and goal-directed actions resembling regular activities. The movements of the whole body were recorded by a real-time computer vision algorithm and provided information on the participants’ motor abilities, such as motor coordination and manual skills. Besides the whole-body motor ability, the motor ability of the main body parts involved in the actions was studied. Increasing the knowledge of the motor abnormalities in a cohort of autistic children may enhance clinicians’ expertise on the motor ability in ASD, as well as support the development of automatic biomarker-based procedures for the early assessment of ASD.

To this purpose, three interactive tasks requiring goal-directed actions were used in the study. These interactive tasks differed in terms of the type and quality of the required movement, involving the motor coordination of the entire body. To assess children’s motor skills in controlled ecological settings resembling everyday life activities, the tasks were presented in virtual reality (VR). Indeed, VR can move everyday life experiences into the laboratory, enhancing the ecological validity of the procedure and prompting realistic responses in users (Wallace et al., 2010; Clemente et al., 2014; Parsons T. D., 2016; Minissi et al., 2021, 2022). The use of VR in the assessment and treatment of ASD is extensive (Parsons S., 2016), and the CAVE-Automatic Virtual Environment (CAVE; Cruz-Neira et al., 1992) is reaching consensus for the autistic population, due to the reduced risk of discomfort (Bowman et al., 2002; Parsons et al., 2004; Wallace et al., 2010; Pastorelli and Herrmann, 2013; Yuan and Ip, 2018; Alcañiz Raya et al., 2020a,b; Minissi et al., 2021, 2022).

Besides testing whether the type of task could modulate the presence of motor abnormalities in children with ASD or whether their manifestation is evident across tasks, regardless of the type of required action, the study hypotheses were (1) children with ASD presented atypical prospective control of fine movements as shown by motor abnormalities in the limbs mainly involved in the action, and (2) the deficit in motor performance in children with ASD affected the motor behavior of the whole body, as described by the atypical movements reported in body parts not directly involved in the action. To test these hypotheses, the whole-body movements were tracked across tasks, and specific motor metrics were computed.

## 2. Methods

### 2.1. Participants

Forty children (20 with TD and 20 with ASD) between 3 and 6 years old participated in the study. The group of children with ASD was recruited at the Development Neurocognitive Centre Red Cenit (Valencia, Spain), and was composed of 16 males and 4 females, according to the prevalence ratio of the disorder (four males every one female diagnosed; Fombonne, 2009). The mean age in months in the autistic group was 53.44 (*SD* = 13.22). In the group, ASD was diagnosed previously by expert and certified clinicians of the Development Neurocognitive Centre Red Cenit, using the Autism Diagnostic Observation Schedule 2 (ADOS-2; Lord et al., 1999). The exclusion criteria of the ASD group were the presence of neurodevelopmental, psychiatric, and anxiety comorbidities. Expert clinicians evaluated the presence of these comorbidities in the ASD group.

The group of children with TD gathered 10 males and 10 females with a mean age in months of 59.40 (*SD* = 11.95). The group was recruited through local study promotion on social media. None of the children with TD presented a previous diagnosis of neurodevelopmental or psychiatric and anxiety disorders. Nevertheless, children’s caregivers were asked to answer a short *ad hoc developed* questionnaire to check the presence of symptoms related to these disorders.

Participants of both groups were Spanish and right-handed, as reported by children’s caregivers. They were drug naïve and had normal or corrected to normal vision. The study participation was voluntary. Caregivers were informed about the study and gave written consent prior to children’s participation. The Ethical Committee of the Polytechnic University of Valencia approved the study (ID: P_06_04_06_20).

### 2.2. Procedure

#### 2.2.1. The virtual reality system

**Figure 1:**
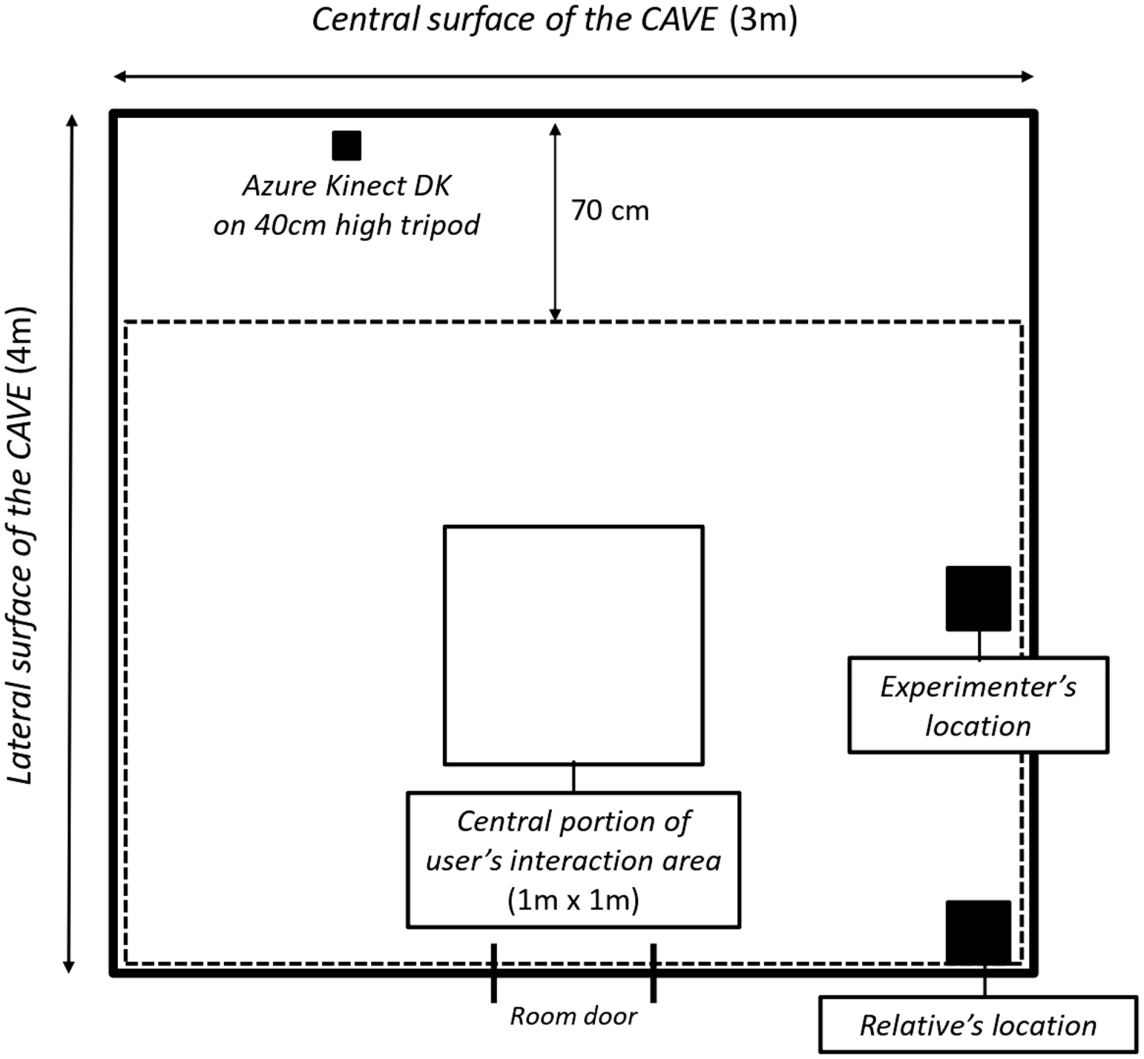
Experimental setting representation. Please note that figure is not scaled according to real dimensions. The dotted line enclosed the user’s interaction area in which participants could interact virtually with the system, and in which the body tracking was guaranteed. The biggest filled-black squares represented the experimenter’s location during the study and the relative’s location (if their presence was required due to participant’s age).

The VR system was a three-surface CAVE with dimensions of 4 m × 4 m × 3 m, and three ultra-short lens projectors positioned in the ceiling, which projected 100° images at 55 cm of distance. The CAVE was located in the Development Neurocognitive Centre Red Cenit in a room with no windows. The virtual environment was projected on the central surface of the CAVE, while the two lateral surfaces remained black. The Azure Kinect DK (Microsoft Corporation, 2019) provided the interaction in the virtual environment. It was set on a 40 cm high tripod in front of the central surface of the CAVE, not interfering with the user’s vision of the virtual environment. The Azure Kinect DK used a depth camera in the resolution mode of 640 × 576 at 30 frames per second. The camera’s depth of field allowed the user’s body tracking in the entire CAVE. Therefore, participants were free to move throughout the entire room, except for the portion of space 70 cm in front of the CAVE central surface, due to user occlusion from the camera (see Figure 1). To provide participants with the optimal user interaction during the task performance, they were encouraged to stay as much as possible in the central portion of the user’s interaction area in the CAVE, which size was 1 m x 1 m (see Figure 1). Nevertheless, even if participants left the central portion of the interaction area, body tracking and virtual interaction were guaranteed as shown by previous technical testing carried out by the team.

#### 2.2.2. The virtual tasks

The two groups had to perform three interactive virtual tasks differing in the type of motor action required: the ball-kicking task (KT), the flower-picking task (FT), and the bubble-blowing task (BT), described below. The task order was randomized and counterbalanced between participants and groups. These tasks were chosen according to previous study evidence on the manifestation of motor abnormalities during the execution of basic and goal-directed actions with the limbs (e.g., Crippa et al., 2015; Alcañiz Raya et al., 2020b). In particular, three goal-directed actions resembling regular activities related to play were implemented in the current tasks. Asking to perform regular activities related to play may have likely led to significant engagement in children. The task actions differed in the main body part involved in the movement (the upper or the lower limbs). The KT asked for movements with the lower limbs, while the FT and BT asked for movements with the upper limbs. It was decided to present two tasks involving the upper limbs because they required moving arms and hands with different degrees of freedom in the movement. The objective was to test whether the degree of freedom in the movement of the upper limbs had a role in the manifestation of motor abnormalities. This objective was related to previous evidence regarding the presence of motor abnormalities in ASD in tasks involving the upper limbs and differing in the degree of freedom of the required movement (e.g., Forti et al., 2011; Crippa et al., 2015; Vabalas et al., 2020; Alcañiz Raya et al., 2020b; Simeoli et al., 2021). The current task features and usability were adapted to the target populations (Minissi et al., 2021, 2022).

**Figure 2:**
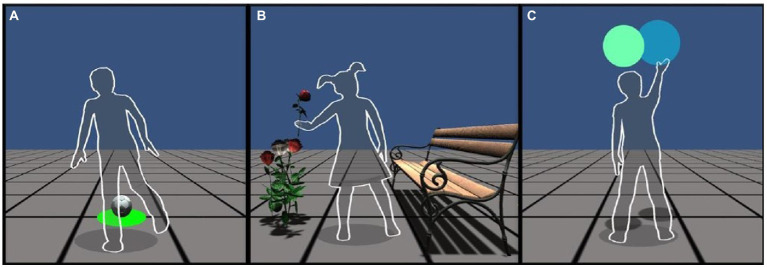
Screen captures of the interactive virtual tasks: **(A)** KT; **(B)** FT; **(C)** BT. Participants could choose to interact through the male or female human shape.

Participants saw in the virtual environment a non-filled virtual human shape mirroring their movements of the head, trunk, and limbs. At the beginning of the study, participants choose between male and female virtual human shape to facilitate personal identification with the avatar and enhance the sense of virtual immersion (see Figure 2).

In the ball-kicking task (KT), participants had to kick a virtual ball for five times (see Figure 2A). It was required to make precise movements with one of the lower limbs to kick the ball. The motor movement of the rest of the body supported the whole-body coordination in the action. At the beginning of the task, participants found the virtual ball in front of the virtual human shape, located in a virtual area easy to reach. To avoid unintentional kicks, the ball appeared in a red shadow and turned green after 3 s, indicating participants they could kick. To avoid irrelevant body displacements, the location of the virtual balls varied depending on the user’s location at the final stage of the previous kick. Thus, participants could always find the virtual ball in front of the virtual human shape.

In the flower task (FT), participants had to move virtual flowers horizontally from a picking-up area on the left side of the virtual space to a leaving area on the right side (the bench; see Figure 2B). The virtual locations of picking and leaving were fixed. The participants had to accomplish the task performing sharp and goal-directed movements with one of their upper limbs. This action was repeated five times. The upper limbs were the central body part involved in the action, and participants had to coordinate them in a visuomotor manner to reach the virtual locations of pick-up and leaving correctly. Consequently, whole-body motor coordination was required to take action correctly.

In the bubble task (BT), participants had to explode falling bubbles by touching and making movements with their upper limbs (see Figure 2C). The bubbles had different colors and fell in pairs vertically in front of the virtual human shape on the central surface of the CAVE. The falling location of the bubbles was random and limited to the portion of the virtual area reachable by the upper limbs of the virtual human shape. Therefore, participants could find the bubbles in many locations and not in predetermined areas. Participants had to touch virtually the bubbles with their upper limbs, involving visuomotor coordination, while the rest of the body supported the reaching of bubble locations. The motor movements required to explode the bubbles were characterized by a high-level degree of freedom because participants could touch the bubbles in different locations and at any fall stage. They also had to coordinate their movements as quickly as possible throughout the CAVE, trying to reach most of the bubbles. The number of falling bubbles was set to 30 instead of 5 as in the previous tasks. This choice was twofold: in accordance with the study on children’s user experience (Minissi et al., 2021, 2022) and to ensure the assessment of the movement variability typical of the actions with high-level degree of freedom. To avoid ceiling and floor effects in the performance and maintaining the engagement, the bubbles had increasing falling speeds (Minissi et al., 2021, 2022): the first 10 were slow (0.20 m/s), the second 10 were moderate (0.30 m/s), and the last 10 were rapid (0.45 m/s).

Before the experimental testing, the child familiarized with the experimenter outside the CAVE and at the presence of their caregivers to reduce the risk of fear and anxiety towards her. Afterwards, there were a few minutes of initial adaptation to the virtual system in which the experimenter invited participants to move their upper/lower limbs to recognize their movements in the virtual human shape. Once participants understood that the virtual human shape mirrored their movements, the experimental testing could start. At the beginning of each task, the experimenter instructed participants on task objectives, using predetermined sentences (i.e., in the KT: “When the ball turns green, you can kick it”; in the BT: “Bubbles are falling, let us explode them!”; in the FT: “Do you see the flowers? Let us take one and bring it on the bench”). Whether participants did not understand the task objective, more in-depth instructions were provided following a top-down strategy: (1) instruction repetition supported by cueing of the virtual target, (2) imitation from the experimenter of the required movement, (3) direct examples of how to perform provided by the experimenter. In-depth instructions were provided progressively only in case participants remained unresponsive for more than 30 s. Finally, at the task beginning, the user’s virtual location was at the center of the virtual environment to reduce the variability in displacements toward targets.

#### 2.2.3. Body tracking and processing

The Azure Kinect DK recorded the body tracking using a real-time computer vision algorithm to obtain the joint positions of the user’s body over time. In each task, motor performance recording of each participant started in the beginning of the first trial. Data related to the instruction phases were excluded for subsequent analyses.

Raw data contained tracking of all people in the camera’s field of view even though they were not interfering with the participant’s virtual interaction. These people were the supervising researcher and, in some cases, the participants’ relatives who were required due to their short age (see Figure 1). Therefore, a manual clean-up was necessary to identify and select the user’s trace. This procedure was carried out by the first and second authors, checking the users’ recordings frame by frame and indicating among the tracked individuals who was the child. Three criteria guided this procedure: (1) the children were performing the task around the central interaction area while the participants’ relatives and the experimenter remained seated close to a room corner and on the side of the room respectively; (2) the children always presented lower height than further tracked people; (3) the children’s limbs had reduced length compared to further people.

**Figure 3:**
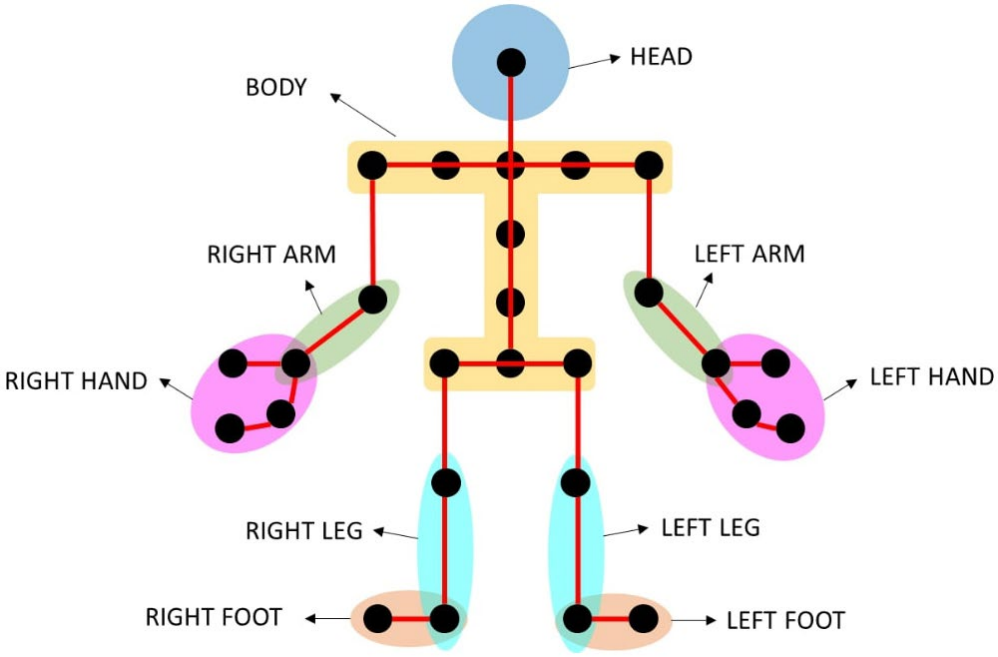
Body joints tracked by the Azure Kinect DK (black dots on the skeleton) and joint groups created (circled in different colors).

Moreover, occasionally parts of the joints were lost in some frames due to occlusions caused by the participant’s body position. Participants missing joints were repaired by interpolating the position of adjacent nodes in time, with a maximum of five consecutive missing joints to be interpolated (average of approximately 0.2 s). Then, the coordinate positions of each joint were smoothed using a multiple-pass moving average filter. A uniform-size window of five samples was used, and a minimum of three samples in the window was selected to avoid a null value. The rolling window was applied five times to achieve smooth position curves. Subsequently, the instantaneous displacement, instantaneous velocity, and instantaneous acceleration were calculated for each joint marked as a black point in Figure 3.

The instantaneous displacement was computed following Eq. (1), which corresponds to the Euclidean distance between two consecutive position points, $r_{n}$ and $r_{n-1}$ , with coordinates (x,y,z) respectively, in time interval *i*. The instantaneous velocity was calculated as the ratio of the displacement to time increment between two consecutive frames. The instantaneous acceleration was obtained by dividing the change in velocity by the time step between consecutive points. A minimum sampling frequency of 10 Hz was established in the metric calculation, eliminating values captured with a time step higher than 0.1 s, which occurred only in 0.54% of the data collected.

(1) $$d_{i}=d_{i}\left(\vec{r_{n}},\vec{r_{n-1}}\right)=\sqrt{{\left(x_{n}-x_{n-1}\right)}^{2}+{\left(y_{n}-y_{n-1}\right)}^{2}+{\left(z_{n}-z_{n-1}\right)}^{2}}$$

Once the instantaneous displacement, velocity and acceleration were computed for each joint, the values for the 10 joint groups (indicated in colors in Figure 3) were obtained by calculating the mean of the available joints for each group. Finally, the following motor metrics were computed for each joint group: the sum of displacement, mean and maximum velocity, mean and maximum of positive accelerations (i.e., accelerations), and mean and maximum of negative accelerations (i.e., decelerations). A threshold of 40% missing samples was set with respect to the total instantaneous values of the task, otherwise the metrics from that joint group were discarded. Moreover, the body tracking data after the manual clean-up step was also used to calculate the duration of the tasks.

### 2.3. Data analysis

Firstly, an independent sample t-test was applied to check that there was no difference in age measured in months between groups. Consequently, comparison tests were applied to assess differences in motor metrics and task duration to test the research hypotheses, comparing children with ASD and TD. Shapiro–Wilk test was applied to determine whether the metrics were normally distributed, and Levene’s test was applied to check the homogeneity of variance.

Group differences were analyzed using the t-test for normal and homogeneous distributions, Welch’s t-test for normal and non-homogeneous distributions, and the Mann–Whitney Wilcoxon test for non-normal distributions. Due to the large amount of computed test (n = 214), the Bonferroni-Holm multicomparison correction was applied, and the significance level was set at α = 0.00024. Eta squared (η2) was reported in the statistically significant results to measure the effect size. Statistical analyses were conducted using the SciPy Python library.

## 3. Results

Participants of the two groups did not differ in age [*t*(38) = −1.506; *p* = 0.140].

**Figure 4:**
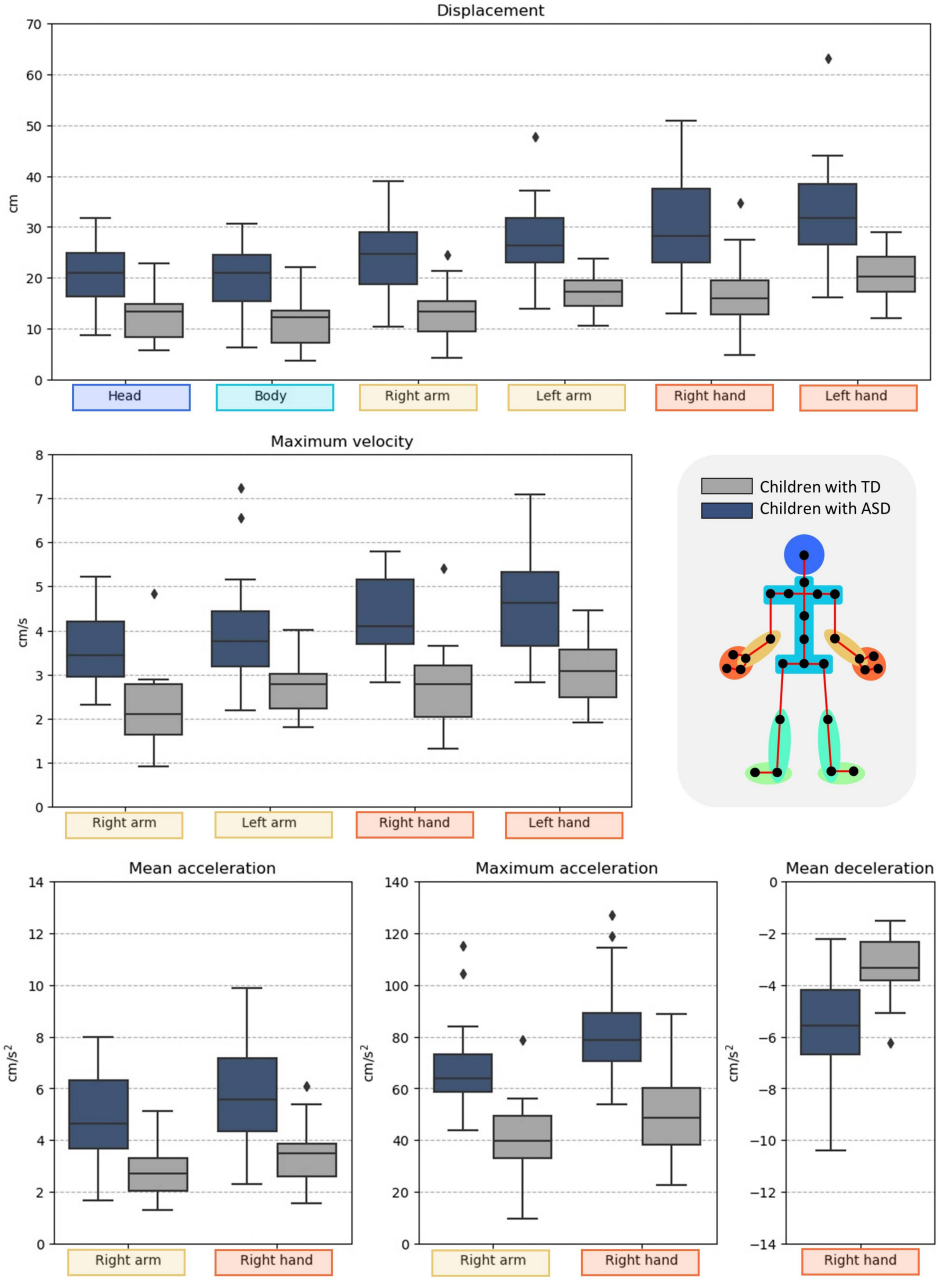
Visual summary of the statistically significant results on motor metrics in the FT.

Regarding the body’s movements in the KT and BT, the two groups did not show differences in their motor behavior (see Supplementary Tables 1, 2 in the Supplementary material). On the contrary, in the FT, the two groups’ motor behavior was different at a significant level. Table 1 shows the motor metrics of the body parts which difference in the FT was statistically significant between groups. The same results are represented in Figure 4. The non-statistically significant results in the FT were reported in the Supplementary Table 3 of the Supplementary material.

**Table 1:** Motor metrics and body parts that were statistically significantly different between children with ASD and TD in the FT.

Motor metric	Joint group	Description	t-statistic	*p*-value	η^2^
Displacement	Head	ASD: 20.394 (6.313) TD: 12.302 (4.404)	*t*(38) = −4.58	<0.0001	1.45
	Body	ASD: 19.813 (6.421) TD: 11.128 (4.541)	*t*(38) = −4.81	<0.0001	1.52
	Right arm	ASD: 24.231 (8.162) TD: 12.821 (5.123)	*t*(37) = −5.06	<0.0001	1.64
	Left arm	ASD: 27.079 (7.903) TD: 16.944 (3.944)	*t*(38) = −5.00	<0.0001	1.58
	Right hand	ASD: 29.583 (10.278) TD: 16.521 (6.74)	*t*(38) = −4.63	<0.0001	1.46
	Left hand	ASD: 32.971 (10.102) TD: 20.759 (4.867)	*t*(38) = −4.75	<0.0001	1.50
Maximum velocity	Right arm^†^	ASD: 3.434 (1.26) TD: 2.098 (1.14)	*t*(37) = 38.0	<0.0001	.80
	Left arm	ASD: 3.999 (1.231) TD: 2.672 (0.564)	*t*(38) = −4.27	0.0002	1.35
	Right hand	ASD: 4.349 (0.865) TD: 2.681 (0.913)	*t*(38) = −5.78	<0.0001	1.83
	Left hand	ASD: 4.585 (1.183) TD: 3.12 (0.743)	*t*(38) = −4.57	0.0001	1.44
Mean acceleration	Right arm	ASD: 4.865 (1.723) TD: 2.827 (1.04)	*t*(36) = −4.23	0.0002	1.41
	Right hand	ASD: 5.746 (1.823) TD: 3.395 (1.119)	*t*(37) = −4.7	0.0001	1.52
Maximum acceleration	Right arm	ASD: 68.166 (18.095) TD: 40.18 (14.678)	*t*(36) = −5.12	<0.0001	1.66
	Right hand	ASD: 82.843 (19.04) TD: 49.383 (15.567)	*t*(37) = −5.86	<0.0001	1.88
Deceleration mean	Right hand	ASD: −5.569 (1.845) TD: −3.307 (1.119)	*t*(37) = 4.53	0.0001	1.45

† This variable was not normally distributed. The median (interquartile range) is detailed in the description.

In the FT, the children with ASD showed more significant head, body, and upper limbs displacement. They also reported more remarkable maximum velocity in the upper limbs and greater mean and maximum acceleration in the right upper limb compared to their peers with TD. In addition, the deceleration mean of the right hand was lower in children with ASD.

Finally, task duration did not differ between groups in the KT and BT [KT: t(38) = 204; *p* = 0.9246; BT: *t*(38) = −0.71; *p* = 0.4857]. In the FT, there was a statistically significant difference in time needed to perform [t(38) = 55.0; *p* = 0.0001; η^2^ = 0.73]. Children with ASD needed more time compared to their TD peers to complete the task (children with ASD: *M* = 96.160 s, *SD* = 65.92 s; children with TD: *M* = 40.992 s, *SD* = 22.86 s).

## 4. Discussion

The present study investigated the motor skills in children with ASD and TD during the execution of three interactive and immersive virtual tasks asking them to perform precise and goal-directed movements. In particular, in the kick task (KT), participants had to move one of their lower limbs, imitating kicking a ball. In the flower task (FT), they had to move accurately one of their upper limbs on the horizontal axis between fixed virtual areas. Finally, in the bubble task (BT), users had to move their upper limbs to reach virtual targets falling in the virtual environment. The target movements in the BT were characterized by a greater degree of freedom than the other two tasks since bubbles fell with increasing speed in random rather than fixed virtual locations. Despite a variety of studies used the CAVE for the autistic population (e.g., Wallace et al., 2010; Tzanavari et al., 2015; Yuan and Ip, 2018; Alcañiz Raya et al., 2020a,b; Minissi et al., 2021, 2022), to our knowledge little attention was given to the study of motor skills in individuals with ASD in tasks requiring virtual interactions. The potential of VR to resemble everyday life activities could be promising in the ecological assessment of motor skills in children with ASD.

The current findings shed light on the presence of motor abnormalities in children with ASD in one task out of three (the FT). Compared to the TD group, participants with ASD presented in this task more significant head, body, and upper limbs displacement. Moreover, they also showed more maximum velocity in the upper limbs, greater mean and maximum acceleration in the right upper limb and lower mean deceleration of the right hand. Similarly, the FT was the only task showing differences in the execution time between groups since children with ASD needed more time on average to perform.

These findings showed that whole-body motor abnormalities in children with ASD have emerged in one specific task asking for precise and goal-directed movements of the upper limbs with a low degree of freedom. These findings are in line with many studies reporting motor abnormalities in individuals with ASD during the execution of tasks requiring precise and goal-directed actions with the upper limbs, like moving real objects in particular locations, imitating circumscribed actions, or dragging virtual elements (e.g., Mari et al., 2003; Forti et al., 2011; Crippa et al., 2015; Anzulewicz et al., 2016; Gowen et al., 2020; Vabalas et al., 2020; Emanuele et al., 2021; Simeoli et al., 2021). The above evidence may suggest that if the purpose is detecting ASD motor abnormalities in goal-directed actions, asking for precise movements of the upper limbs could be more distinctive than asking for movements with a greater degree of freedom involving arms and hands (as in the BT), or precise movements of other parts of the body (as in the KT). The lack of motor differences between groups in the KT and the BT reinforced this idea. It may be related to several potential factors: first, the different types of actions required by these tasks; second, the familiarity with these actions that children may have compared to the FT; and third, the level of individual engagement in the KT and BT.

However, while a large portion of ASD studies focused on the assessment of circumscribed motor skills in the upper limbs, to our knowledge, not many studies have investigated goal-directed motor actions with a greater degree of freedom or precise movements with further parts of the body (i.e., the feet, the head, and the body). Therefore, it could be that scientific interest in testing precise and goal-directed movements of the upper limbs in individuals with ASD (and consequently the increasing number of published studies on the topic) may have promoted this hypothesis instead of further possibilities.

Regarding the motor abnormalities in ASD that emerged in the study, the first hypothesis of the current research was that children with ASD would present atypical prospective control of fine movements, as shown by the presence of motor abnormalities in the limb mainly involved in the action. The results showed that in the FT, the motor abnormalities were particularly evident in the upper limbs, which movement was required to move the virtual flowers between the picking up and leaving locations. As can be seen, even though participants were right-handed, the findings were significantly different in both upper limbs. Indeed, not all participants picked the virtual flowers with their right hand, and some decided to pick with their left hand (see an example in Figure 2B). This choice might be twofold: the proximity between the left virtual hand and the picking-up location, which was closer to the flowers than the right virtual hand, and the fact that hand dominance is not entirely stable at that age (Hildreth, 1949). In this view, the current findings on the upper limbs in the FT may favor our hypothesis that children with ASD would present motor abnormalities in the body part mainly involved in the required action. In particular, the more significant displacement in the upper limbs of children with ASD may reflect excessive movement when they have to perform precise and goal-directed actions. Indeed, children with TD accomplished the task efficiently without needing the same amount of displacement in the arms and hands. This finding confirmed previous studies reporting more significant displacement and excessive movements in individuals with ASD in their upper limbs and other parts of the body (Glazebrook et al., 2009; Chang et al., 2010; Martin et al., 2018; Alcañiz Raya et al., 2020b; Zhao et al., 2022). The excessive motor movements may underly the attempt of autistic children to adjust their motor trajectory progressively to achieve the goal of the action, performing more fragmented and unprecise movements (Cook et al., 2013; Whyatt and Craig, 2013; Simeoli et al., 2021). This progressive motor adjustment in individuals with ASD is likely related to their atypical prospective control of movements (Klin et al., 2003; Cattaneo et al., 2007; Trevarthen and Delafield-Butt, 2013). Indeed, the present findings are in line with Fabbri-Destro et al. (2009), which suggested impaired ability in ASD to transform the cognitive motor planning into effective execution. Consequently, the progressive adjustment of the movement may drive the cognitive perception of autistic individuals about their motor actions, which are perceived as divided into many independent submovements rather than as part of a unique entity.

Besides the more significant displacement, the children with ASD presented greater maximum velocity, mean and maximum accelerations in the upper limbs and a more significant mean deceleration in the right hand. This confirmed previous studies reporting different velocity and acceleration/deceleration patterns in the arms and hands of children with ASD during the execution of goal-directed actions requiring the manipulation of natural objects (e.g., Forti et al., 2011; Crippa et al., 2015; Vabalas et al., 2020). Similarly, greater velocities and accelerations in the hands of children with ASD have been found during the execution of fine movements on digital devices (e.g., tablets; Anzulewicz et al., 2016; Simeoli et al., 2021). In the tasks of the previous studies and the FT, the oculomotor control of the precise and goal-directed movements of the arms and hands (i.e., eye-hand coordination) was critical to undertake the required actions correctly. According to Crippa et al. (2015) and Glazebrook et al. (2009), autistic individuals tended to present inefficient eye-hand coordination due to the asynchronicity between the ocular processes and the motor behavior. Such oculomotor inefficiency has also been reported in tasks involving 3D user interfaces (Mei et al., 2014, 2015). Due to inefficient eye-hand coordination, children with ASD may present motor abnormalities in the upper limbs that their atypical prospective control of movements can intensify.

Taken together, these findings fostered the hypothesis of the manifestation of ASD motor abnormalities when arms and hands are the body part mainly involved in the precise and goal-directed action to perform. This is consistent with Crippa et al. (2015) and Simeoli et al. (2021), that claimed kinematic analysis of simple upper-limb movement might reliably identify ASD in a cohort of children.

Contrary to this hypothesis, one study reported some efficiency in object control skills in male children with ASD (Berkeley et al., 2001). Besides the different methodology and nature of the tasks involved in this study (virtual versus natural object manipulation), a possible reason to explain this difference in findings lay in the ASD sample involved. Berkeley et al. (2001) studied object control skills in children with ASD characterized by a low level of symptom severity, while the autistic participants of the current study were selected without considering their level of symptom severity. Thus, it is likely to find different patterns of performance when comparing autistic children with a low level of symptom severity to children across the entire range of ASD (Jarrold and Brock, 2004).

The second hypothesis of the current study was that deficits in motor performance in children with ASD might affect the motor behavior of the whole body, as described by the atypical movements reported in body parts not directly involved in the action to be accomplished. The present findings favored this hypothesis because in the FT, which required specific movements of the upper limbs, children with ASD reported more significant displacement in the head and the body compared to their peers with TD. These findings were reported in addition to the motor differences in the upper limbs described above. The atypical movements in the head and the body of autistic individuals have been previously found in tasks not asking directly to accomplish motor actions with these body parts (e.g., Ardalan et al., 2019; Alcañiz Raya et al., 2020b; Zhao et al., 2022). These abnormal movements suggested that deficits in the cognitive transition from motor planning to the execution of the movement could be extended to body parts not directly involved in the circumscribed action demanded. Therefore, the motor deficit in autistic individuals might be generalized, affecting the motor coordination of the entire body rather than localized on specific body parts (Ardalan et al., 2019; Zampella et al., 2021). In this case, this idea seemed to be plausible particularly when it is required to perform precise and goal-directed actions with the upper limbs.

Finally, children with ASD needed more time to perform and accomplish the task than their peers with TD. The task duration was computed taking into account only the amount of time in which children performed their motor movements. Hence, it reflected previous findings of longer movement durations in children and adolescents with ASD (Mari et al., 2003; Glazebrook et al., 2009; Forti et al., 2011; Crippa et al., 2015; Minissi et al., 2021, 2022). It seems that individuals with ASD are characterized by generalized motor slowness and need more time to prepare their movements and transform their motor intention into operation when the task difficulty is great (Mari et al., 2003; Glazebrook et al., 2009). The different duration of the motor actions had a role in the difference in the motor metric of displacement. Indeed, they mutually influence each other directly, strengthening the idea that children with ASD need more time to plan precise and goal-direct actions and execute them due to the progressive adjustment of their motor trajectory.

In summary, when the interest is studying the motor skills of individuals with ASD while performing goal-directed actions, it seems that the type of required movement could modulate the manifestation of the motor abnormalities. In particular, the motor tasks asking for precise and goal-directed movements of the upper limbs with a low degree of freedom (as the FT) could be more distinctive for the manifestation of the motor abnormalities than other tasks based on different types of goal-directed actions (such as the KT and the BT). Concerning the further hypotheses of this study, (1) the motor abnormalities in children with ASD were evident in the body part mainly involved in the target action, and (2) in the body parts not directly related to the action demanded, indicating that motor abnormalities cover the motor coordination of the whole body. These findings fostered the idea that, in specific tasks, autistic children are characterized by inefficient eye-hand coordination and atypical prospective control of movements due to impairments in transforming motor planning into effective execution. Studies such as the present one contributed to a profound knowledge of motor abnormalities in ASD. The current findings may address the development of virtual interactive procedures for the early motor assessment of the disorder, involving objective and implicit measures and ecological settings.

### 4.1. Limitations and future directions

The study presented some limitations regarding the sample and the experimental setting that are worth to be mentioned. In particular, some autistic participants could not interact with the VR system due to their severe symptomatology. Only the children (with or without ASD) who felt comfortable in the experimental setting, and understood the virtual interaction and task mechanics (following the instruction guidelines in the Procedure section) were included, reaching the sample size of 40 children. In addition, the two groups of participants were balanced for chronological age, but they were not IQ-matched. There are further instances of previous studies in which samples were not IQ-matched (e.g., Crippa et al., 2015; Zhao et al., 2022); nevertheless, measuring IQ would have given information regarding the manifestation of the motor abnormalities in ASD. In particular, there might be a negative relation between IQ and the severity level of motor abnormalities (Green et al., 2009). Future studies are encouraged to assess IQ and investigate this possible relation, fractioning into severity ranges the motor abnormalities measured by quantitative methods. Likewise, it could be of scientific interest studying the relationship between ASD symptom severity and the manifestation of motor abnormalities. This may improve the stratification of ASD based on quantitative methods. Furthermore, the two groups were not balanced for gender. Although gender imbalance between groups was controlled to represent the gender ratio of the studied populations, future studies in which gender is balanced between groups are encouraged to assess whether the present results are reliable even in a group of children with ASD equally composed by male and females. In addition, there is a need to run more studies on ASD motor behavior in different tasks to confirm the current vision that precise and goal-directed movements of the upper limbs characterized by a low degree of freedom may be particularly challenging for individuals with ASD compared to further movements. Future studies are encouraged to measure the motor skills of ASD children in a variety of goal-directed movements, assessing also the effect of the direction of the movement (i.e., rightward as in the FT, or leftward). These future studies should also involve larger sample sizes. Likewise, it could be of scientific interest comparing the motor skills of several young clinical populations (e.g., children with attention and hyperactivity disorder, and children with developmental coordination disorder) on this precise and goal-directed movements of arms and hands to disentangle whether it is a specific motor signature of ASD.

Finally, the experimental setting suffered from tracking limitations related to the overlap between the user’s interaction area and the experimenter’s location. This overlap led to the manual clean-up for identifying and selecting the user’s trace (see the Procedure section), reducing the automatization of data processing. In the studies that the research group is currently carrying out, care and advanced technical solutions have been implemented to capture and track only the participant’s body.

## Data availability statement

The raw data supporting the conclusions of this article will be made available by the authors, without undue reservation.

## Ethics statement

The Ethical Committee of the Polytechnic University of Valencia approved the study (ID: P_06_04_06_20). Written informed consent to participate in this study was provided by the participants’ legal guardian/next of kin.

## Author contributions

MM, FM, MS, MA, and IC contributed to conception and design of the study. MM carried out the experiment. LG-Z and JM-M performed the statistical analysis. MM and LG-Z wrote the first draft of the manuscript. All authors contributed to the article and approved the submitted version.

## Funding

This work was supported by the Spanish Ministry of Economy, Industry, and Competitiveness-funded project “T-EYE: “Monitoring system for children with ASD based on artificial intelligence and physiological measures” (IDI-20201146), and by the project funded by the Ministry of Science and Innovation of Spain ADAPTEA (PID2020-116422RB-C21). It was also co-founded by the European Union through the Operational Program of the European Regional development Fund (FEDER) of the Valencian Community 2014–2020 (IDIFEDER/2018/029 and IDIFEDER/2021/038). Furthermore, MM received funding by the Valencian Community (GRISOLIAP/2019/137 and CIBEFP/2021/38), and by the Polytechnic University of Valencia (Predoctoral Mobility Grant of 2021). LG-Z received partial funding from the Valencian Community (ACIF/2021/187). JM-M received partial funding from the Polytechnic University of Valencia (PAID-10-20).

## Conflict of interest

The authors declare that the research was conducted in the absence of any commercial or financial relationships that could be construed as a potential conflict of interest.

## Publisher’s note

All claims expressed in this article are solely those of the authors and do not necessarily represent those of their affiliated organizations, or those of the publisher, the editors and the reviewers. Any product that may be evaluated in this article, or claim that may be made by its manufacturer, is not guaranteed or endorsed by the publisher.
