# Supplementary material for: The whole-body motor skills of children with autism spectrum disorder taking goal-directed actions in virtual reality
Source: Front Psychol. 2023 Apr 6;14:1140731. doi: 10.3389/fpsyg.2023.1140731 (PMC10117537; doi:10.3389/fpsyg.2023.1140731)
Supplement: Supplementary file 1 [file Table_1.docx]

**Table 1**: Non-statistically significant results between groups regarding the motor metrics in the KT. † This variable was not normally distributed. The median (interquartile range) is detailed in the description.

| Motor metric | Joint group | Description | t-statistic | p-value |
| --- | --- | --- | --- | --- |
| Displacement | Head | ASD: 14.174 (5.512)  TD: 12.96 (5.299) | t(38) = 0.71 | 0.4928 |
|  | Body | ASD: 13.655 (5.431)  TD: 11.064 (5.325) | t(38) = 1.52 | 0.3112 |
|  | Right arm | ASD: 22.988 (8.743)  TD: 11.864 (5.325) | t(38) = 0.59 | 0.5684 |
|  | Left arm | ASD: 22.992 (9.139)  TD: 23.77 (11.579) | t(38) = 0.24 | 0.8195 |
|  | Right hand | ASD: 34.192 (14.368)  TD: 33.866 (16.368) | t(38) = 0.55 | 0.5922 |
|  | Left hand | ASD: 31.799 (13.41)  TD: 36.836 (15.781) | t(38) = 1.08 | 0.6726 |
|  | Right leg | ASD: 15.462 (6.68)  TD: 20.998 (8.15) | t(38) = 2.35 | 0.1308 |
|  | Left leg | ASD: 16.451 (4.862)  TD: 15.27 (8.379) | t(38) = 0.55 | 0.5991 |
|  | Right foot | ASD: 15.373 (5.46)  TD: 22.049 (8.58) | t(38) = 274.0 | 0.0468 |
|  | Left foot | ASD: 16.677 (4.539)  TD: 17.254 (8.985) | t(38) = 0.26 | 0.8045 |
| Mean acceleration | Head† | ASD: 3.42 (1.619)  TD: 1.876 (0.944) | t(38) = -3.59 | 0.0009 |
|  | Body | ASD: 3.339 (2.13)  TD: 1.643 (1.65) | t(38) = 74.0 | 0.0007 |
|  | Right arm | ASD: 4.058 (3.45)  TD: 2.54 (1.96) | t(38) = 100.0 | 0.0071 |
|  | Left arm† | ASD: 4.578 (2.05)  TD: 2.686 (1.453) | t(38) = -3.28 | 0.0022 |
|  | Right hand | ASD: 4.666 (3.76)  TD: 3.04 (2.09) | t(38) = 103.0 | 0.0090 |
|  | Left hand† | ASD: 5.484 (2.485)  TD: 3.515 (1.953) | t(38) = -2.72 | 0.0099 |
|  | Right leg† | ASD: 4.012 (1.975)  TD: 2.47 (1.124) | t(38) = -2.96 | 0.0053 |
|  | Left leg† | ASD: 3.694 (1.631)  TD: 1.962 (1.294) | t(38) = -3.63 | 0.0008 |
|  | Right foot† | ASD: 4.155 (2.1)  TD: 2.675 (1.309) | t(38) = -2.61 | 0.0130 |
|  | Left foot† | ASD: 3.75 (1.652)  TD: 2.067 (1.445) | t(38) = -3.34 | 0.0019 |
| Mean deceleration | Head† | ASD: -3.248 (1.405)  TD: -1.778 (0.913) | t(38) = 3.82 | 0.0005 |
|  | Body† | ASD: -3.127 (1.383)  TD: -1.642 (0.885) | t(38) = 3.94 | 0.0003 |
|  | Right arm | ASD: -3.837 (2.98)  TD: -2.461 (2.15) | t(38) = 302.0 | 0.0060 |
|  | Left arm† | ASD: -4.355 (1.861)  TD: -2.612 (1.475) | t(38) = 3.20 | 0.0028 |
|  | Right hand | ASD: -4.579 (3.38)  TD: -2.904 (2.35) | t(38) = 300.0 | 0.0071 |
|  | Left hand† | ASD: -5.326 (2.383)  TD: -3.411 (1.954) | t(38) = 2.71 | 0.0101 |
|  | Right leg† | ASD: -3.918 (1.952)  TD: -2.368 (1.158) | t(38) = 2.98 | 0.0051 |
|  | Left leg† | ASD: -3.585 (1.547)  TD: -1.895 (1.268) | t(38) = 3.68 | 0.0007 |
|  | Right foot† | ASD: -4.123 (2.172)  TD: -2.628 (1.328) | t(38) = 2.56 | 0.0145 |
|  | Left foot | ASD: -3.714 (2.48)  TD: -2.032 (2.23) | t(38) = 304.0 | 0.0051 |
| Maximum acceleration | Head† | ASD: 48.895 (20.148)  TD: 34.538 (17.507) | t(38) = -2.35 | 0.0244 |
|  | Body† | ASD: 48.118 (21.447)  TD: 31.538 (18.341) | t(38) = -2.56 | 0.0145 |
|  | Right arm† | ASD: 64.116 (20.007)  TD: 42.89 (19.696) | t(38) = -3.30 | 0.0021 |
|  | Left arm | ASD: 161.272 (95.74)  TD: 110.411 (99.07) | t(38) = 134.0 | 0.0382 |
|  | Right hand | ASD: 76.112 (31.7)  TD: 47.186 (22.37) | t(38) = 103.0 | 0.0090 |
|  | Left hand | ASD: 156.952 (104.62)  TD: 103.492 (80.58) | t(38) = 131.0 | 0.0319 |
|  | Right leg | ASD: 73.928 (70.97)  TD: 104.02 (83.75) | t(38) = 130.0 | 0.0301 |
|  | Left leg† | ASD: 63.184 (20.095)  TD: 41.108 (24.029) | t(38) = -3.07 | 0.0039 |
|  | Right foot | ASD: 81.465 (46.38)  TD: 139.604 (114.73) | t(38) = 95.0 | 0.0024 |
|  | Left foot† | ASD: 68.389 (17.969)  TD: 47.491 (28.727) | t(38) = -2.69 | 0.0113 |
| Maximum deceleration | Head | ASD: -0.002 (0.01)  TD: -0.001 (0.001) | t(38) = 0.31 | 0.2124 |
|  | Body | ASD: -0.002 (0.001)  TD: -0.003 (0.001) | t(38) = 121.0 | 0.0169 |
|  | Right arm | ASD: -0.003 (0.01)  TD: -0.009 (0.02) | t(38) = 1.20 | 0.0226 |
|  | Left arm | ASD: -0.006 (0.01)  TD: -0.008 (0.01) | t(38) = 0.63 | 0.0455 |
|  | Right hand | ASD: -0.011 (0.02)  TD: -0.009 (0.01) | t(38) = 0.40 | 0.1897 |
|  | Left hand | ASD: -0.002 (0.001)  TD: -0.012 (0.01) | t(38) = 121.0 | 0.0860 |
|  | Right leg | ASD: -0.005 (0.01)  TD: -0.002 (0.001) | t(38) = 274.0 | 0.0468 |
|  | Left leg | ASD: -0.001 (0.001)  TD: -0.002 (0.01) | t(38) = 0.45 | 0.4091 |
|  | Right foot | ASD: -0.005 (0.01)  TD: -0.006 (0.01) | t(38) = 1.90 | 0.3676 |
|  | Left foot | ASD: -0.005 (0.01)  TD: -0.007(0.01) | t(38) = 0.63 | 0.3084 |
| Maximum velocity | Head† | ASD: 2.682 (1.17)  TD: 1.85 (0.911) | t(38) = -2.44 | 0.0192 |
|  | Body† | ASD: 2.631 (1.229)  TD: 1.716 (0.955) | t(38) = -2.56 | 0.0145 |
|  | Right arm† | ASD: 3.381 (0.968)  TD: 2.34 (1.11) | t(38) = -3.08 | 0.0038 |
|  | Left arm | ASD: 3.191 (0.975)  TD: 2.387 (1.11) | t(38) = -2.37 | 0.0228 |
|  | Right hand† | ASD: 4.027 (1.103)  TD: 2.891 (1.251) | t(38) = -2.97 | 0.0051 |
|  | Left hand | ASD: 11.488 (6.47)  TD: 9.677 (5.6) | t(38) = 134.0 | 0.0382 |
|  | Right leg | ASD: 7.673 (5.29)  TD: 8.943 (3.37) | t(38) = 0.91 | 0.1685 |
|  | Left leg† | ASD: 3.315 (1.099)  TD: 2.263 (1.305) | t(38) = -2.69 | 0.0106 |
|  | Right foot | ASD: 8.893 (3.36)  TD: 12.013 (5.06) | t(38) = 96.0 | 0.0026 |
|  | Left foot† | ASD: 3.658 (1.081)  TD: 2.571 (1.515) | t(38) = -2.55 | 0.0155 |
| Mean velocity | Head | ASD: 0.327 (0.117)  TD: 0.294 (0.122) | t(38) = 0.87 | 0.4089 |
|  | Body | ASD: 0.311 (0.112)  TD: 0.269 (0.121) | t(38) = 1.13 | 0.2695 |
|  | Right arm | ASD: 0.564 (0.213)  TD: 0.595 (0.283) | t(38) = 0.39 | 0.7079 |
|  | Left arm | ASD: 0.554 (0.228)  TD: 0.549 (0.267) | t(38) = 0.06 | 0.9530 |
|  | Right hand | ASD: 0.826 (0.317)  TD: 0.868 (0.39) | t(38) = 0.37 | 0.7169 |
|  | Left hand | ASD: 0.769 (0.335)  TD: 0.785 (0.38) | t(38) = 0.14 | 0.8920 |
|  | Right leg | ASD: 0.447 (0.157)  TD: 0.467 (0.182) | t(38) = 0.37 | 0.7104 |
|  | Left leg | ASD: 0.413 (0.132)  TD: 0.356 (0.179) | t(38) = 1.14 | 0.2758 |
|  | Right foot | ASD: 0.496 (0.198)  TD: 0.544 (0.213) | t(38) = 0.73 | 0.4858 |
|  | Left foot | ASD: 0.457 (0.154)  TD: 0.412 (0.199) | t(38) = 0.80 | 0.4389 |

**Table 2**: Non-statistically significant results between groups regarding the motor metrics in the BT. † This variable was not normally distributed. The median (interquartile range) is detailed in the description.

| Motor metric | Joint group | Description | t-statistic | p-value |
| --- | --- | --- | --- | --- |
| Displacement | Head | ASD: 29.751 (11.518)  TD: 27.447 (10.104) | t(38) = 0.67 | 0.5160 |
|  | Body | ASD: 28.88 (11.636)  TD: 24.747 (9.863) | t(38) = 1.21 | 0.2449 |
|  | Right arm | ASD: 52.557 (18.899)  TD: 59.168 (20.952) | t(38) = 1.05 | 0.3136 |
|  | Left arm† | ASD: 48.098 (27.08)  TD: 50.53 (30.67) | t(38) = 0.22 | 0.3115 |
|  | Right hand | ASD: 74.751 (28.304)  TD: 88.299 (29.732) | t(38) = 1.55 | 0.1585 |
|  | Left hand | ASD: 68.748 (28.134)  TD: 78.103 (31.646) | t(38) = 1.07 | 0.3490 |
|  | Right leg | ASD: 30.925 (12.928)  TD: 26.895 (11.691) | t(38) = 1.09 | 0.3261 |
|  | Left leg | ASD: 29.145 (11.541)  TD: 25.496 (9.999) | t(38) = 1.20 | 0.3042 |
|  | Right foot | ASD: 31.099 (13.035)  TD: 27.444 (12.089) | t(38) = 1.01 | 0.4034 |
|  | Left foot | ASD: 28.568 (11.082)  TD: 26.239 (9.892) | t(38) = 0.63 | 0.5038 |
| Mean acceleration | Head† | ASD: 3.337 (1.646)  TD: 1.853 (0.894) | t(38) = -3.45 | 0.0017 |
|  | Body† | ASD: 3.209 (1.637)  TD: 1.682 (0.81) | t(38) = -3.64 | 0.0011 |
|  | Right arm† | ASD: 4.717 (1.947)  TD: 3.409 (1.265) | t(38) = -2.46 | 0.0196 |
|  | Left arm† | ASD: 8.737 (5.63)  TD: 8.556 (5.69) | t(38) = 0.11 | 0.4608 |
|  | Right hand† | ASD: 16.186 (7.61)  TD: 17.103 (8.22) | t(38) = 0.42 | 0.4386 |
|  | Left hand† | ASD: 12.237 (7.99)  TD: 13.351 (9.08) | t(38) = 0.37 | 0.4165 |
|  | Right leg† | ASD: 3.276 (1.818)  TD: 1.65 (0.675) | t(37) = -3.57 | 0.0017 |
|  | Left leg† | ASD: 3.146 (1.665)  TD: 1.588 (0.658) | t(38) = -3.80 | 0.0008 |
|  | Right foot† | ASD: 3.231 (1.741)  TD: 1.65 (0.66) | t(36) = -3.52 | 0.0020 |
|  | Left foot† | ASD: 2.989 (1.584)  TD: 1.585 (0.615) | t(37) = -3.52 | 0.0018 |
| Mean deceleration | Head† | ASD: -3.128 (1.508)  TD: -1.746 (0.868) | t(38) = 3.46 | 0.0016 |
|  | Body† | ASD: -3.042 (1.532)  TD: -1.577 (0.777) | t(38) = 3.72 | 0.0009 |
|  | Right arm† | ASD: -4.455 (1.81)  TD: -3.273 (1.276) | t(38) = 2.33 | 0.0254 |
|  | Left arm† | ASD: -8.509 (5.8)  TD: -7.829 (4.81) | t(38) = 0.42 | 0.3840 |
|  | Right hand† | ASD: -8.687 (4.17)  TD: -8.718 (3.92) | t(37) = 0.001 | 0.7215 |
|  | Left hand† | ASD: -13.035 (8.43)  TD: -12.882 (8.27) | t(37) = 0.39 | 0.4608 |
|  | Right leg† | ASD: -3.145 (1.711)  TD: -1.575 (0.627) | t(37) = 3.67 | 0.0013 |
|  | Left leg† | ASD: -2.992 (1.61)  TD: -1.523 (0.629) | t(38) = 3.70 | 0.0011 |
|  | Right foot† | ASD: -3.134 (1.675)  TD: -1.593 (0.614) | t(36) = 3.58 | 0.0017 |
|  | Left foot† | ASD: -2.926 (1.57)  TD: -1.531 (0.605) | t(37) = 3.53 | 0.0018 |
| Maximum acceleration | Head† | ASD: 50.093 (28.94)  TD: 32.594 (25.93) | t(38) = 2.05 | 0.0137 |
|  | Body | ASD: 49.024 (33.18)  TD: 35.019 (18.42) | t(38) = 116.0 | 0.0239 |
|  | Right arm† | ASD: 162.993 (104.34)  TD: 135.958 (88.33) | t(37) = 0.90 | 0.1162 |
|  | Left arm | ASD: 156.31 (87.009)  TD: 127.528 (63.17) | t(37) = 1.12 | 0.2836 |
|  | Right hand† | ASD: 273.916 (175.0)  TD: 208.439 (145.01) | t(37) = 1.27 | 0.0957 |
|  | Left hand† | ASD: 192.261 (137.47)  TD: 165.718 (134.74) | t(37) = 0.65 | 0.1525 |
|  | Right leg | ASD: 78.345 (46.99)  TD: 40.392 (28.9) | t(37) = 114.0 | 0.0339 |
|  | Left leg† | ASD: 82.597 (63.55)  TD: 69.608 (72.37) | t(37) = 0.67 | 0.0701 |
|  | Right foot | ASD: 85.607 (53.75)  TD: 45.383 (25.95) | t(36) = 90.0 | 0.0089 |
|  | Left foot† | ASD: 92.203 (48.87)  TD: 67.245 (46.86) | t(36) = 1.64 | 0.0825 |
| Maximum deceleration | Head | ASD: -0.001 (0.001)  TD: -0.001 (0.001) | t(38) = 0.0001 | 0.9999 |
|  | Body | ASD: -0.002 (0.001)  TD: -0.002 (0.001) | t(38) = 0.0001 | 0.9999 |
|  | Right arm† | ASD: -0.005 (0.01)  TD: -0.003 (0.01) | t(37) = 0.62 | 0.2823 |
|  | Left arm† | ASD: -0.003 (0.001)  TD: -0.004 (0.01) | t(37) = 0.43 | 0.1108 |
|  | Right hand† | ASD: -0.004 (0.01)  TD: -0.004 (0.01) | t(37) = 0.003 | 0.2919 |
|  | Left hand† | ASD: -0.005 (0.01)  TD: -0.003 (0.01) | t(37) = 0.62 | 0.1276 |
|  | Right leg | ASD: -0.002 (0.001)  TD: -0.001 (0.01) | t(37) = 0.43 | 0.1460 |
|  | Left leg† | ASD: -0.002 (0.001)  TD: -0.001 (0.01) | t(37) = 0.43 | 0.0562 |
|  | Right foot | ASD: -0.001 (0.001)  TD: -0.001 (0.01) | t(36) = 0.001 | 0.9999 |
|  | Left foot† | ASD: -0.003 (0.001)  TD: -0.001 (0.01) | t(36) = 6.24 | 0.1637 |
| Maximum velocity | Head | ASD: 2.694 (1.48)  TD: 1.92 (0.89) | t(36) = 105.0 | 0.0106 |
|  | Body | ASD: 2.769 (1.4)  TD: 1.827 (0.86) | t(38) = 100.0 | 0.0071 |
|  | Right arm† | ASD: 13.91 (13.31)  TD: 11.078 (4.92) | t(38) = 0.96 | 0.0538 |
|  | Left arm† | ASD: 10.548 (14.12)  TD: 10.922 (6.52) | t(37) = 0.0001 | 0.1335 |
|  | Right hand† | ASD: 21.894 (18.64)  TD: 17.07 (9.01) | t(38) = 134.0 | 0.0380 |
|  | Left hand† | ASD: 15.843 (12.79)  TD: 16.051 (21.56) | t(37) = 0.18 | 0.0739 |
|  | Right leg† | ASD: 7.682 (7.25)  TD: 9.288 (7.55) | t(37) = 0.89 | 0.3215 |
|  | Left leg† | ASD: 3.388 (1.371)  TD: 2.56 (0.938) | t(38) = -2.17 | 0.0360 |
|  | Right foot | ASD: 3.861 (2.47)  TD: 2.271 (0.9) | t(38) = 110.0 | 0.0422 |
|  | Left foot | ASD: 7.833 (4.26)  TD: 6.552 (4.57) | t(38) = 120.0 | 0.0250 |
| Mean velocity | Head | ASD: 0.354 (0.151)  TD: 0.308 (0.106) | t(38) = 1.24 | 0.2908 |
|  | Body | ASD: 0.337 (0.149)  TD: 0.275 (0.098) | t(38) = 1.61 | 0.1348 |
|  | Right arm† | ASD: 0.708 (0.35)  TD: 0.742 (0.31) | t(38) = 0.33 | 0.3180 |
|  | Left arm† | ASD: 0.575 (0.36)  TD: 0.586 (0.33) | t(37) = 0.12 | 0.2116 |
|  | Right hand† | ASD: 0.929 (0.46)  TD: 1.1 (0.49) | t(38) = 1.30 | 0.1897 |
|  | Left hand† | ASD: 0.796 (0.52)  TD: 0.862 (0.47) | t(38) = 0.42 | 0.1881 |
|  | Right leg | ASD: 0.394 (0.183)  TD: 0.312 (0.119) | t(38) = 1.70 | 0.1106 |
|  | Left leg | ASD: 0.378 (0.167)  TD: 0.292 (0.105) | t(38) = 1.93 | 0.0658 |
|  | Right foot | ASD: 0.426 (0.196)  TD: 0.326 (0.126) | t(34) = 2.37 | 0.0979 |
|  | Left foot | ASD: 0.381 (0.161)  TD: 0.308 (0.106) | t(38) = 1.66 | 0.1128 |

**Table 3**: Non-statistically significant results between groups regarding the motor metrics in the FT. † This variable was not normally distributed. The median (interquartile range) is detailed in the description.

| Motor metric | Joint group | Description | t-statistic | p-value |
| --- | --- | --- | --- | --- |
| Displacement | Right leg† | ASD: 16.359 (6.21)  TD: 9.361 (4.649) | t(38) = -3.93 | 0.0003 |
|  | Left leg† | ASD: 17.516 (6.171)  TD: 10.287 (5.046) | t(38) = -3.95 | 0.0003 |
|  | Right foot† | ASD: 14.6 (6.364)  TD: 9.281 (4.546) | t(33) = -2.80 | 0.0085 |
|  | Left foot† | ASD: 15.476 (5.949)  TD: 10.377 (5.121) | t(36) = -2.76 | 0.0090 |
| Mean acceleration | Head | ASD: 3.281 (2.13)  TD: 2.046 (1.22) | t(37) = 105.0 | 0.0176 |
|  | Body | ASD: 3.295 (2.05)  TD: 1.837 (1.24) | t(38) = 95.0 | 0.0047 |
|  | Left arm† | ASD: 5.102 (1.697)  TD: 3.611 (1.228) | t(38) = -3.10 | 0.0036 |
|  | Left hand† | ASD: 6.102 (1.933)  TD: 4.403 (1.396) | t(38) = -3.10 | 0.0036 |
|  | Right leg | ASD: 3.381 (1.91)  TD: 1.86 (1.17) | t(38) = 100.0 | 0.0071 |
|  | Left leg | ASD: 3.542 (2.24)  TD: 1.948 (1.25) | t(38) = 100.0 | 0.0071 |
|  | Right foot | ASD: 4.068 (1.856)  TD: 3.007 (1.878) | t(38) = 103.0 | 0.0450 |
|  | Left foot | ASD: 3.366 (2.0)  TD: 2.184 (1.52) | t(35) = 104.0 | 0.0459 |
| Mean deceleration | Head† | ASD: -3.54 (1.527)  TD: -2.318 (1.017) | t(37) = 2.88 | 0.0066 |
|  | Body† | ASD: -3.461 (1.503)  TD: -2.076 (1.014) | t(38) = 3.33 | 0.0021 |
|  | Right arm† | ASD: -4.616 (1.729)  TD: -2.764 (1.022) | t(36) = 3.86 | 0.0007 |
|  | Left arm† | ASD: -4.887 (1.695)  TD: -3.287 (1.029) | t(38) = 3.52 | 0.0011 |
|  | Left hand | ASD: -5.723 (2.09)  TD: -4.408 (2.36) | t(38) = 318.0 | 0.0015 |
|  | Right leg† | ASD: -3.535 (1.742)  TD: -2.059 (1.238) | t(38) = 3.01 | 0.0046 |
|  | Left leg | ASD: -3.354 (2.1)  TD: -1.781 (1.2) | t(38) = 294.0 | 0.0114 |
|  | Right foot† | ASD: -3.449 (1.976)  TD: -2.193 (1.31) | t(33) = 2.19 | 0.0356 |
|  | Left foot† | ASD: -3.44 (1.78)  TD: -2.43 (2.74) | t(36) = 1.65 | 0.0153 |
| Maximum acceleration | Head | ASD: 57.369 (18.38)  TD: 37.487 (16.84) | t(37) = 81.0 | 0.0023 |
|  | Body | ASD: 61.318 (22.16)  TD: 33.919 (19.53) | t(38) = 83.0 | 0.0016 |
|  | Left arm | ASD: 70.77 (23.31)  TD: 53.712 (15.51) | t(38) = 70.0 | 0.0005 |
|  | Left hand | ASD: 79.754 (34.12)  TD: 63.375 (20.1) | t(38) = 65.0 | 0.0003 |
|  | Right leg | ASD: 66.321 (22.14)  TD: 40.06 (24.0) | t(38) = 87.0 | 0.0023 |
|  | Left leg† | ASD: 78.185 (33.761)  TD: 43.225 (15.16) | t(38) = -4.12 | 0.0003 |
|  | Right foot† | ASD: 67.167 (22.989)  TD: 48.46 (26.128) | t(33) = -2.14 | 0.0397 |
|  | Left foot | ASD: 69.259 (29.65)  TD: 49.943 (20.73) | t(35) = 65.0 | 0.0014 |
| Maximum deceleration | Head† | ASD: -0.0001 (0.01)  TD: -0.0001 (0.01) | t(38) = 54.0 | 0.0481 |
|  | Body | ASD: -0.002 (0.001)  TD: -0.005 (0.01) | t(38) = 1.34 | 0.0568 |
|  | Right arm† | ASD: -0.003 (0.01)  TD: -0.009 (0.02) | t(35) = 1.20 | 0.0226 |
|  | Left arm | ASD: -0.002 (0.01)  TD: -0.006 (0.01) | t(35) = 0.80 | 0.1092 |
|  | Right hand | ASD: -0.009 (0.01)  TD: -0.006 (0.01) | t(35) = 0.60 | 0.2538 |
|  | Left hand | ASD: -0.012 (0.02)  TD: -0.005 (0.01) | t(35) = 1.40 | 0.0599 |
|  | Right leg | ASD: -0.003 (0.01)  TD: -0.005 (0.01) | t(35) = 0.63 | 0.1806 |
|  | Left leg† | ASD: -0.004 (0.01)  TD: -0.002 (0.01) | t(35) = 0.63 | 0.3733 |
|  | Right foot† | ASD: -0.004 (0.01)  TD: -0.003 (0.01) | t(35) = 0.32 | 0.2155 |
|  | Left foot | ASD: -0.001 (0.01)  TD: -0.003 (0.01) | t(35) = 0.63 | 0.0782 |
| Maximum velocity | Head | ASD: 3.084 (1.26)  TD: 1.954 (1.06) | t(38) = 72.0 | 0.0006 |
|  | Body† | ASD: 3.26 (1.406)  TD: 1.937 (0.745) | t(38) = -3.62 | 0.0011 |
|  | Right leg† | ASD: 3.475 (1.229)  TD: 2.28 (1.054) | t(38) = -3.22 | 0.0026 |
|  | Left leg† | ASD: 3.977 (1.466)  TD: 2.432 (0.824) | t(38) = -4.0 | 0.0003 |
|  | Right foot† | ASD: 3.54 (1.122)  TD: 2.623 (1.191) | t(33) = -2.24 | 0.0318 |
|  | Left foot† | ASD: 4.185 (1.545)  TD: 2.883 (0.91) | t(36) = -3.12 | 0.0036 |
| Mean velocity | Head† | ASD: 0.35 (0.17)  TD: 0.318 (0.18) | t(38) = 0.73 | 0.2452 |
|  | Body | ASD: 0.34 (0.15)  TD: 0.269 (0.17) | t(38) = 1.58 | 0.1552 |
|  | Right arm† | ASD: 0.637 (0.29)  TD: 0.503 (0.25) | t(38) = 1.52 | 0.0739 |
|  | Left arm | ASD: 0.628 (0.171)  TD: 0.587 (0.185) | t(38) = 0.73 | 0.4811 |
|  | Right hand† | ASD: 0.865 (0.29)  TD: 0.702 (0.25) | t(38) = 1.87 | 0.0405 |
|  | Left hand | ASD: 0.841 (0.23)  TD: 0.785 (0.241) | t(38) = 0.81 | 0.4672 |
|  | Right leg | ASD: 0.439 (0.165)  TD: 0.374 (0.216) | t(38) = 1.19 | 0.3032 |
|  | Left leg† | ASD: 0.433 (0.25)  TD: 0.287 (0.22) | t(38) = 2.01 | 0.1092 |
|  | Right foot | ASD: 0.444 (0.189)  TD: 0.418 (0.233) | t(38) = 0.42 | 0.7310 |
|  | Left foot† | ASD: 0.417 (0.23)  TD: 0.329 (0.27) | t(38) = 1.10 | 0.2553 |
